# Prof. Frank Donaldson, M. D., on Air and Ventilation

**Published:** 1881-06

**Authors:** 


### PROF. FRANK DONALSON, M.D., ON AIR AND VENTILATION.

---

A man by his breath will spoil in 24 hours, about 350 cubic feet of air. He ought to have 1000 cubic feet of space and the air ought to be renewed three times per hour.

Every human being, every pet animal, in a house, consumes the air; and when once thus used, it becomes unfit for further use, because it contains effete substances, the result of the wear and tear of the organism. These when rebreathed enter the lungs, and with the air penetrate into the blood, and thence pass to the structure of the body. The exhaled air contains less oxygen by nearly one-fourth than the inspired column; thus every breath takes away from the really nutrive element of the air. The tissues suffer primarily from deficient supply of oxygen. An adult man exhales on an average 6 cubic feet of carbonic acid per hour. The limit of the maximum impurity that can be borne is .7 carbonic acid per 1000 volumes. According to Dr. Carpenter, an adult man gives off 160 grains of carbon per hour. Dr. Parkes gives the average amount of carbonic acid exhaled by an adult in the twenty-four hours as sixteen cubic feet.

The air as it is expired is heated. Hartley states the bodily heat of one man will cause a rise of temperature of 150 cubic feet of air, equal to 173 degrees. Hence the great heating effect of a mass of human beings packed closely together in badly ventilated and ill-lighted buildings.

The expired air contains ammonia in appreciable quantity. The inmates of the famous Black Hole of Calcutta suffered from it. One of the sufferers described the intolerable irritation caused by the inspired air as though the face was held over hartshorn. Of the one hundred and forty-six persons immured, in two hours fifty were dead. The next morning only twenty-three remained alive, and nearly the whole of these suffered from putrid typhus fever, of which many died. We know the irritating influence of ammonia. According to Dr. Richardson, it tends to hold the blood in a state of fluidity. It also interferes with the process of oxidation of organic matter, so that it becomes an antiseptic, and it rapidly decomposes ozone. The breath and the skin further give off compounds of sulphur and ammonia, sulphide of ammonia, which is directly poisonous. The quantity of water vapor exhaled is estimated at from 25 to 40 ounces in the 24 hours, and requires an average of 210 feet of air per hour to retain it. This vapor is loaded with organic matter, which is especially deleterious to health. It has a very fœtid smell when it is accumulated and but slowly oxidised. It is believed (Prof. Wilson) to be molecular, and may be said to hang around a room like cloud of tobacco smoke Its odor is difficult to get rid of, even after free ventilation. It darkens sulphuric acid and discolorises solutions of permanganate of potash. When put in pure water it becomes offensive. In sick-rooms it is associated with pus-cells and other emanations of disease.

Prof. Remsen correctly stated that the carbonic acid by itself is not poisonous. It exists in our tissues and circulates in our blood. He should have added, *that it has been demonstrated that the amount of organic matter in air vitiaed by respiration is found to increase as the carbonic acid increases.* Dr. Parkes states, and others have confirmed his statement, that it becomes perceptible to the sense of smell when the carbonic acid in an inhabited room amounts to 7 per 1,000 cubic feet of air. Tyndall has shown the amount of this organic dust, which is nothing more or less than organic poison, everywhere present indoors. The philanthropist, John Howard, stated that his clothes became so offensive from prison air that he could not ride in coaches, but had to travel on horseback. The leaves of his memorandum book were so tainted that he had first to spread them open for an hour or two before an open fire before he could use them. Such are the emanations of effete matter which are constantly being given off from organised beings and their excreta. These exhalations, though of a highly putrescent character, especially during disease, and escaping into the atmosphere, under ordinary circumstances, are not very cognizable by the senses, except when given off as one or other of the numerous family of volatile alkaline principles. In cold climates like that of Russia, a process goes on during the prolonged winter which exhibits very clearly the large amount of organic matter diffused in human exhalations. The moisture given off by the lungs and skins of the imprisoned inmates of the dwellings of the lower orders in the north of Russia is gradually condensed and frozen on the inside of the windows, where it is often allowed to remain, undergoing a change of putrefaction (septic fermentation). On the approach of summer, the general thaw causes the melting of this deposit, and thereby setting free the products of decomposition, gives rise to certain most offensive odors, for which the abodes of Russian peasantry, at the breaking of the ice, are notorious. (Condy) So charged with this azotised matter is the air which we expire, that if we breathe into a jug of water, putrefactive decomposition is readily perceived. Dr. R. Angus Smith has estimated the quantity as 3 per 1,000. It is to impurities of this nature that we attribute contagion and infection. It settles in houses, and gets into carpets and woolen materials, and in niches amid dust and dirt.

Thus, my friends, we show you that even if our breaths were the only means of rendering the air of our houses impure that it would be unfit for health unless renewed incessantly, but we have always at work other destroyers of air.

Our means of illumination by gas destroys an immense quantity of the air of houses, and leaves injurious products of combustion. One cubic foot of coal-gas destroys the oxygen of eight cubic feet of air in combustion, and produces about two cubic feet of carbonic acid, besides other impurities. A common gas-burner burns about three cubic feet of gas per hour. How long can the air remain pure when there are present several persons, each consuming sixty gallons of it per hour, with gas-jets each destroying as much air as eleven men—660 gallons per hour? If stoves are used, the consumption of air is fearful—not less than 15,000 gallons of air per hour. *It is impossible to breathe, or have fires or lights, without robbing air of its oxygen and loading it with impurities.* When Dalton (as given by Lewis) analysed the air of a room in which during two hours fifty candles had been burning and five hundred persons breathing, he found that instead of the proportion of carbonic acid being only two gallons in five thousand of air, it was not less than one gallon in every hundred. Leblanc analysed the atmosphere of three hospitals in Paris, and found that they contained respectivelv five, ten, and twelve times as much carbonic acid as the air of the streets, and organic matter in proportion. We acknowledge that we look forward with confidence and great pleasure to the substitution of the electric light, as now produced by Edison and others, for gas. It will be a great improvement in lighting our churches, theatres and dwellings. With it there are three great advantages: It does not *consume the air*, for it is produced in *vacuums* by incandescent carbon points; it gives out *no heat;* and there is no danger from fire, for the instant the globes are broken and air enters, the light goes out. There will be no need of parlor matches for children to play with!

Let us see if there are not other sources of contamination of the air. Frequently we have in houses, from the combustion of coal and charcoal, the *oxide of carbon*, which is a deadly gas, with its characteristic blue flame. It renders the blood corpuscles red, and interferes with the absorption of oxygen. If we breathe this gas, even in very small quantities, it is poisonous. No ventilation will prevent us from being poisoned if we are in a room where coke or charcoal fumes are allowed to escape. Dr. Marye and Mr. Lewis both give instances of fatal asphyxia from this cause, although windows were open and air was circulating. Our ordinary gas, escaping into the air of rooms, contaminates ; fortunately for us, its smell is unmistakable when it is only one part in a thousand ; it becomes very offensive when it is 1-750 or 1-500.

